# The Leloir Cycle in Glioblastoma: Galactose Scavenging and Metabolic Remodeling

**DOI:** 10.3390/cancers13081815

**Published:** 2021-04-10

**Authors:** Martyn A. Sharpe, Omkar B. Ijare, David S. Baskin, Alexandra M. Baskin, Brianna N. Baskin, Kumar Pichumani

**Affiliations:** 1 Kenneth R. Peak Brain and Pituitary Tumor Treatment Center, Department of Neurosurgery, Houston Methodist Neurological Institute, Houston Methodist Hospital and Research Institute, Houston, TX 77030, USA; oijare@houstonmethodist.org (O.B.I.); dbaskin@houstonmethodist.org (D.S.B.); ambaskin@aggienetwork.com (A.M.B.); bbaskin001@utexas.edu (B.N.B.); 2 Department of Neurological Surgery, Weill Cornell Medical College, New York, NY 10065, USA

**Keywords:** GBM, Glut14, galactose scavenging, Leloir pathway, pentose phosphate pathway

## Abstract

**Simple Summary:**

Proliferation of glioblastoma (GBM) depends on access to extracellular nutrients. Inadequate tumor perfusion creates a short supply of the most common nutrients, such as glucose (Glc) and glutamine. As a result, GBMs undergo metabolic remodeling and scavenge alternative nutrients from the tumor microenvironment for their growth and proliferation. GBM expresses sugar transporters, such as Glut3 and Glut14. Galactose (Gal) is a good substrate for Glut3/Glut14, and GBM cells can scavenge Gal from the circulation/extracellular space. The Leloir pathway allows GBM to transport and metabolize Gal at physiologic Glc concentrations, providing GBM cells with an alternate energy source. GBM cultures proliferated when grown solely on Gal. GBMs metabolize Gal via the Leloir pathway, glycolysis, and pentose phosphate pathway (PPP) to optimize ATP production, while the mitochondrial metabolism of Gal in GBM is limited. The selective targeting of the Leloir/PPP pathway may provide new treatment strategies for GBM.

**Abstract:**

Background: Glioblastoma (GBM) can use metabolic fuels other than glucose (Glc). The ability of GBM to use galactose (Gal) as a fuel via the Leloir pathway is investigated. Methods: Gene transcript data were accessed to determine the association between expression of genes of the Leloir pathway and patient outcomes. Growth studies were performed on five primary patient-derived GBM cultures using Glc-free media supplemented with Gal. The role of Glut3/Glut14 in sugar import was investigated using antibody inhibition of hexose transport. A specific inhibitor of GALK1 (Cpd36) was used to inhibit Gal catabolism. Gal metabolism was examined using proton, carbon and phosphorous NMR spectroscopy, with ^13^C-labeled Glc and Gal as tracers. Results: Data analysis from published databases revealed that elevated levels of mRNA transcripts of SLC2A3 (Glut3), SLC2A14 (Glut14) and key Leloir pathway enzymes correlate with poor patient outcomes. GBM cultures proliferated when grown solely on Gal in Glc-free media and switching Glc-grown GBM cells into Gal-enriched/Glc-free media produced elevated levels of Glut3 and/or Glut14 enzymes. The ^13^C NMR-based metabolic flux analysis demonstrated a fully functional Leloir pathway and elevated pentose phosphate pathway activity for efficient Gal metabolism in GBM cells. Conclusion: Expression of Glut3 and/or Glut14 together with the enzymes of the Leloir pathway allows GBM to transport and metabolize Gal at physiological glucose concentrations, providing GBM cells with an alternate energy source. The presence of this pathway in GBM and its selective targeting may provide new treatment strategies.

## 1. Introduction

Glioblastoma (GBM) is a grade IV aggressive glial neoplasm, with an incidence in the United States of 3.19 per 100,000 per year. Patient outcome in GBM remains poor, with a median survival of 16.7 months at diagnosis and only 30% of patients surviving two years [1]. Many human cancers, including brain tumors, primarily metabolize glucose (Glc) to lactate for ATP production, a phenomenon known as the Warburg effect [2,3,4]. Recently it has become apparent that malignant brain tumors also catabolize a wide range of circulating nutrients, including fatty acids, ketone bodies, amino acids, and acetate [2,5,6,7]. Glut3 (SLC2A3) is the major transporter of Glc in GBM, with its elevated levels indicative of poor patient outcomes [8,9,10,11,12]. Glut14 (SLC2A14), primarily expressed in primate testicular tissue at higher levels, is a Glut3 paralogue that shares >96% amino acid homology and identical hexose transport kinetics [13,14]. As galactose (Gal) is a good substrate for both Glut3 and Glut14, we postulated that Glut3 or Glut14 may allow GBM cells to scavenge plasma Gal [15,16,17,18].

**Figure 1:**
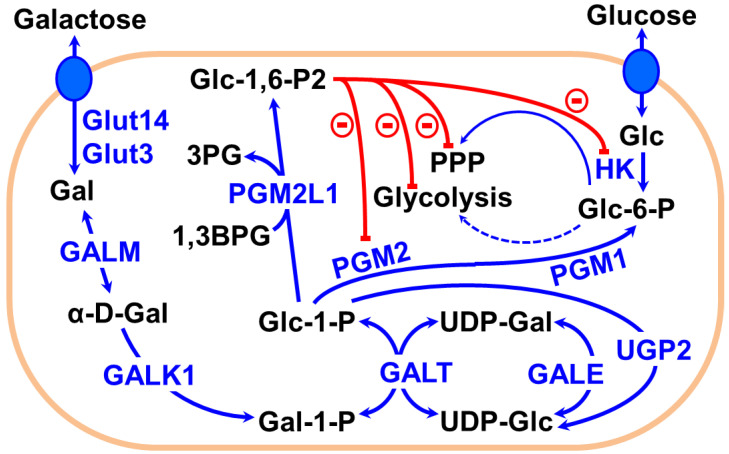
Illustration of the metabolism of galactose through the Leloir pathway. Galactose (Gal) is converted to Gal-1-P by the action of the enzymes GALM and GALK1. Gal-1-P is converted into Glc-1-P by GALT/GALE. Furthermore, Glc-1-P is converted to Glc-6-P (by PGM1 and PGM2), which follows the glycolytic pathway similar to glucose metabolism. Glc-1-P can also be converted to Glc-1,6-P2 (by PGM2L1), which is a master allosteric controller that can inhibit HK (glycolysis) and 6PGGDH (PPP).

Utilization of Gal requires Leloir pathway enzymes, typically found in high levels in the liver, kidney and the gut [19]. Luis Leloir won the Nobel Prize for his work on the Gal oxidation pathway that now bears his name (Figure 1) [20]. Gal is only efficiently imported into cells by either Glut3 or Glut14 and requires an epimerase (GALM) to generate the correct anomer (α-Gal), which is phosphorylated by galactokinase-1 (GALK1) into Gal-1-P. Further, Gal-1-P is converted into Glc-1-P through the combined action of a uridyltransferase (GALT) and UDP-galactose-4-epimerase (GALE), involving the intermediates UDP-Gal and UDP-Glc. UDP-Glc is regenerated by UDP-glucose pyrophosphorylase 2 (UGP2) from Glc-1-P. Phosphoglucomutases (PGM1 and PGM2) convert Glc-1-P into Glc-6-P, which is then metabolized through glycolysis. In the brain, another isoform of phosphoglucomutase (PGM2L1) converts Glc-1-P into Glc-1,6-P2, using 1,3-bisphosphoglycerate (1,3BPG) as co-substrate [21]. Glc-1,6-P2 is a master allosteric controller known to inhibit hexokinase (HK) in glycolysis, 6-phosphogluconate dehydrogenase (6PGDH) in the pentose phosphate pathway (PPP) and fructose-1,6-bisphosphatase (FBP1) in gluconeogenesis (Figure 1) [22,23,24,25,26].

In this study, we characterized the expression of Glut3 and Glut14 in five primary patient-derived GBM cells and showed that GBMs use Gal as an alternative source of energy, utilizing both the Leloir and the PPP pathways. Efficient utilization of Gal by GBM may open up new treatment strategies using Gal-based antimetabolites.

## 2. Results

A visualization of the transcript levels of the Gal transporters, Glut3/Glut14, and key enzymes of the Leloir pathway, stratified by patient outcome, is shown as a heatmap in Supplemental Figure S1A. High levels of transcripts for SLC2A3 and SLC2A14, which efficiently transport Gal, and the Leloir pathway enzymes correlate with poorer patient outcomes. In contrast, expression of PGM2L1 in GBM correlates with improved patient outcome. Using healthy volunteers, Gannon, Khan, and Nuttall measured the plasma Gal levels after ingestion of a Gal-rich meal [27]. We performed kinetic modeling of their data and the analysis shows that dietary Gal uptake is rapid (t_½_ of ≈ 14 min) and the Gal is present in the plasma at a concentration >1 mM for extended periods, but the metabolism of Gal is slow (t_½_ of ≈ 23 min) (Supplemental Figure S1B) [27].

**Figure 2:**
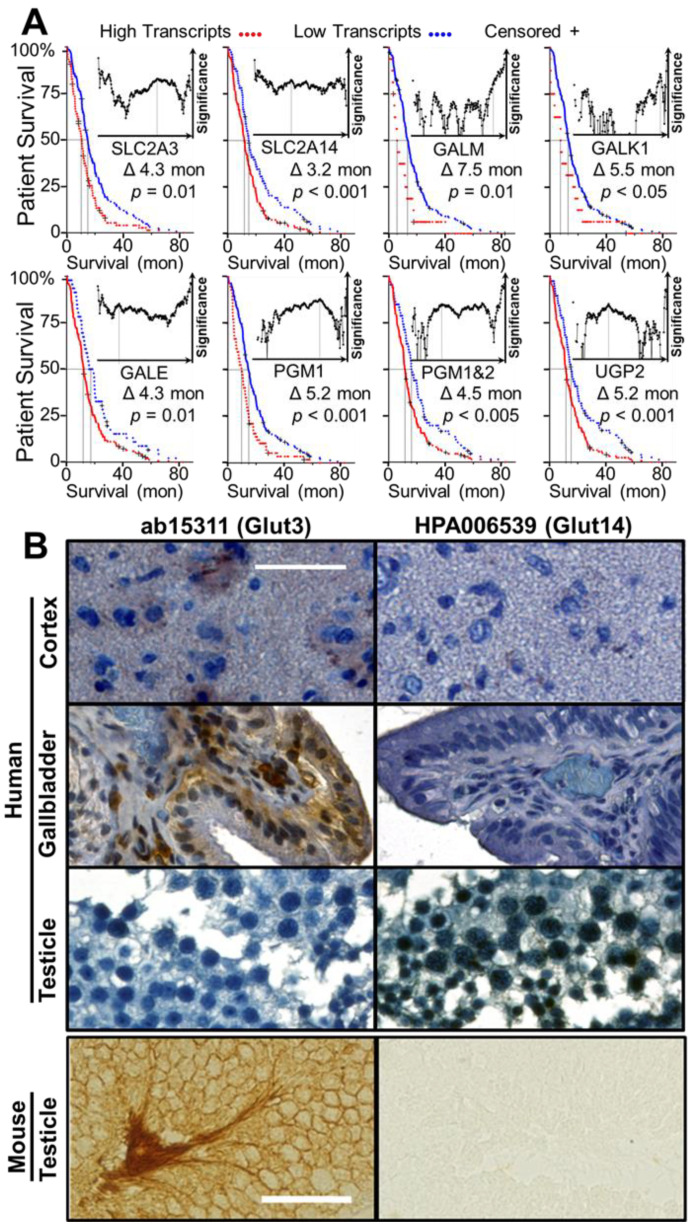
Correlation between the expression levels of Gal import genes (SLC2A3, SLC2A14, GALM, GALK1, GALE, PGM1/2 and UGP2) and the patient survival. Also shown are the expression of Glut3 and Glut14 in human/mouse tissues. (**A**) Kaplan–Meier survival curves of the genes associated with Gal import and metabolism using the TCGA RNA-Seq database: red, high expression; blue, low expression; +, censored. (**B**) ab15311 (anti-Glut3) and HPA006539 (anti-Glut14) and antibody target validation in human and mouse tissues, visualized using DAB staining. Scale bar = 50 µm.

An examination of patient tumor transcriptomes indicates that the ability of GBM to import and catabolize Gal is inversely proportional to patient survival. The impact of expression of Glut3/Glut14 and of other key enzymes of the Leloir pathway on the survival of GBM patients is presented in the form of Kaplan–Meier survival curves (Figure 2A). As Glut14 is known to be restricted to testicular tissue, we examined the expression of SLC2A3 and SLC2A14 in GBM tissue from males and females and observed GBM expresses Glut14 with no significant sex difference in the means, medians, or distribution of either transcript (Supplemental Figure S1C). A high degree of correlation between the transcript levels of SLC2A3 and SLC2A14 was apparent (Supplemental Figure S1D). In the larger TCGA-GBM Agilent 4502A dataset, we found expression of SLC2A14 is inversely proportional to patient survival, *p* < 0.05, and SLC2A3 and SLC2A14 expression is highly correlated (Supplemental Figure S2A) [27,28,29]. Transcript levels of the Leloir pathway enzymes were also inversely proportional to patient survival in the TCGA-GBM Agilent-4502A, Rembrandt [30], and Gravendeel [31] databases, with the sole exception of GALT in the Agilent-4502A-derived dataset (Supplemental Figure S2B), showing no inverse relationship to patient survival.

We wished to use a pair of Glut3-specific and Glut14-specific antibodies for a series of labeling and transport inhibition studies but were unable to find a commercially available antibody that has specificity toward Glut14. Images hosted on the Protein Atlas Database (www.proteinatlas.org, accessed on 16 February 2021) suggested that the nominally Glut3-specific antibody HPA006539 has much greater specificity toward Glut14 than to Glut3, as this antibody labeled Glut14-rich testicular tissue, but not Glut3-rich tissues, such as brain and gallbladder [32,33]. To test the affinity of this antibody, we used both human (accessed from biorepository of Houston Methodist through the approved IRB protocol and the details are given in the Methods section) and mouse testicular tissues. Glut3-specific ab15311 showed labelling in human cerebral cortex and gallbladder glandular cells, but HPA006539 did not show any labeling (Figure 2B). In contrast, Glut14-rich human testicular tissue was strongly labeled by HPA006539, whereas it showed weak labelling by ab15311. Mouse testicular tissue, which does not express Glut14, was used as final control and was strongly labeled by ab15311, but not by HPA006539, concluding that ab15311 has high specificity for Glut3 while HPA006539 for Glut14.

**Figure 3:**
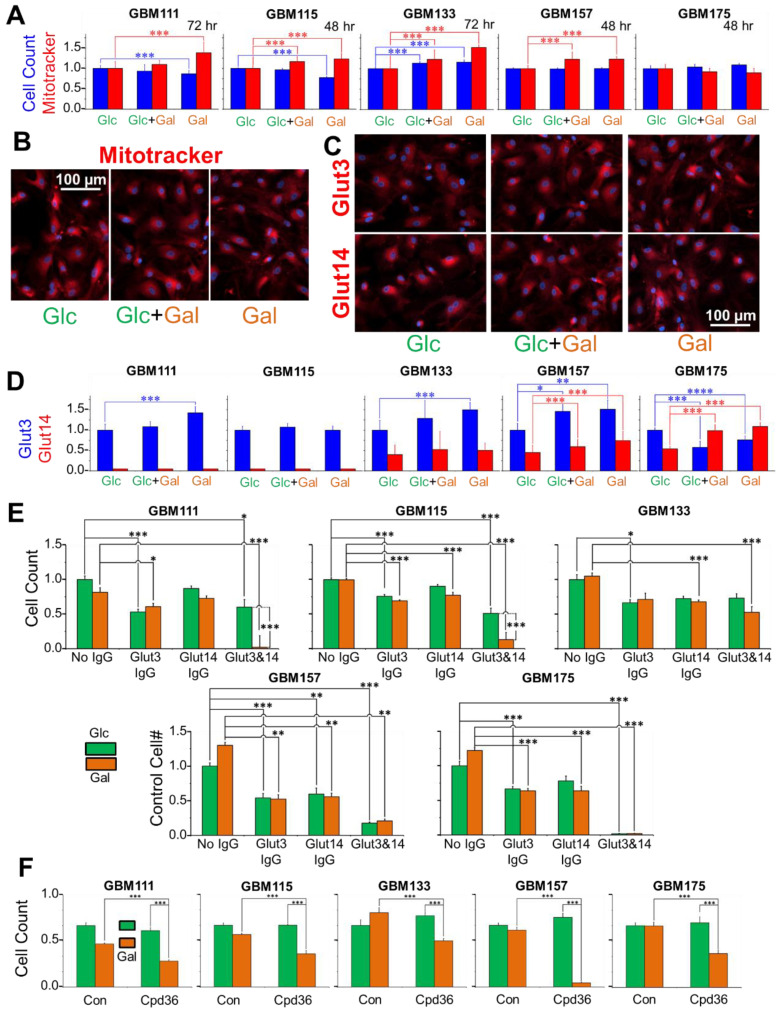
Five primary cell cultures proliferate when grown on Glc and Gal hexose mixtures, altering the mitochondria (**A**, incubation time indicated, *n* = 8; blue bars indicate cell count and red bars indicate Mitotracker intensity), with representative images of the Mitotracker labeling of GBM175 cells (**B**, scale bar 10 µm; red: Mitotracker staining, blue: DAPI staining). Representative images of the ab15311 (anti-Glut3) and/or HPA006539 (anti-Glut14) antibody labeling levels in GBM175 cells (**C**, scale bar 10 µm; red: Glut3 or Glut14 staining, blue: DAPI) and levels of transporters of five primary cell cultures when grown on Glc and Gal hexose mixtures (**D**, *n* = 6; blue bars: Glut3 levels, blue bars: Glut14 levels). Five primary cell cultures grown on Glc and Gal hexose mixtures for 24 h in the presence of a 1/150 dilution of ab15311 (anti-Glut3) and/or HPA006539 (anti-Glut14 (**E**, *n* = 6; green bars: Glc treatment, orange bars: Gal treatment). Five primary cell cultures were incubated for 24 h in 10 µM CDP36, a GALK1-specific inhibitor, and underwent a loss in viability only in Gal-containing, and not Glc-containing, media (**F**, *n* = 6; green bars: Glc treatment, orange bars: Gal treatment), * *p* < 0.05, ** *p* < 0.001, *** *p* < 0.005 and **** *p* < 0.0001.

For cell proliferation studies, all GBM cells (GBM111, GBM115, GBM133, GBM157, and GBM175) were initially grown in Glc media before they were transferred to the new media containing either 4 mM Glc, 2 mM Glc plus 2 mM Gal, or with only 4 mM Gal, and then incubated for the doubling time of each culture. The normalized changes in cell numbers, obtained from cell counts, and the levels of Mitotracker fluorescence per cell of GBM cell lines are shown in Figure 3A, and representative Mitotracker images of GBM175 are shown in Figure 3B. All cell lines were completely viable and proliferated when grown with either Glc, Glc plus Gal, or solely with Gal. Growth of GBM111 and GBM115 was slightly slowed down, whereas growth of GBM133 and GBM175 was slightly increased when Gal was the only hexose present in the media. Mitotracker signals increased in four of the five cell lines (GBM111, GBM115, GBM133, and GBM157) when grown on Glc plus Gal and/or Gal alone (Figure 3A).

Levels of Glut3 and Glut14 were quantified using fluorescence microscopy and the representative images are shown for GBM175 cells in Figure 3C. The plots of the expression levels of Glut3 and Glut14 of all the five cell lines are shown in Figure 3D. GBM111 and GBM115 had high levels of Glut3, but low levels of Glut14 (near the detection threshold). GBM133 and GBM157 expressed relatively higher levels of Glut3 and moderate levels of Glut14 whereas GBM175 expressed moderate levels of both transporters, Glut3 and Glut14. When grown on Glc and Gal mixtures, these cells gave rise to a complex series of changes in the expression levels of Glut3/Glut14. In GBM115 grown on Gal, there were no changes observed in the Glut3 or Glut14 levels. In GBM157, we observed that the Glut3/Glut14 levels increased with increasing Gal, whereas in GBM175 the responses of Glut3/Glut14 were discordant, with Glut14 rising and Glut3 falling.

As shown earlier, antibody binding to Glut transporters slows down hexose transport kinetics, we performed further growth studies utilizing Glc and Gal media in the absence and presence of ab15311 (anti-Glut3) and HPA006539 (anti-Glu14) antibodies [34]. In GBM111 and GBM115, which have low levels of Glut14, growth on Glc was slowed down by anti-Glut3 and unaffected by anti-Glut14, but there was a synergistic effect in the presence of both antibodies. Growth on Gal was also slowed by both anti-Glut3 and anti-Glut14, but the presence of both antibodies was lethal (Figure 3E). Growth in GBM157, supported by either hexose, was slowed in the presence of either antibody (ab15311 or HPA006539). When both hexose transporters are blocked by antibodies, cell numbers collapsed, in either media. Examining GBM175 cells, inhibition of hexose import by either antibody arrests growth in Gal or Glc media, and combination of both antibodies proved to be lethal. These antibody studies clearly demonstrated that ab15311 and HPA006539 target Glut3 and Glu14, respectively, and inhibit both transporters, impacting GBM cells grown on Gal to a greater extent than on Glc (Figure 3E). In addition, we also tested the effect of the GALK1 inhibitor, Cpd36, on the growth of GBM cells [35]. The presence of Cpd36 had no impact on cells grown on Glc, but reduced the viability of cells grown on Gal, indicating the usage of the Leloir pathway by GBM cells (Figure 3F).

**Figure 4:**
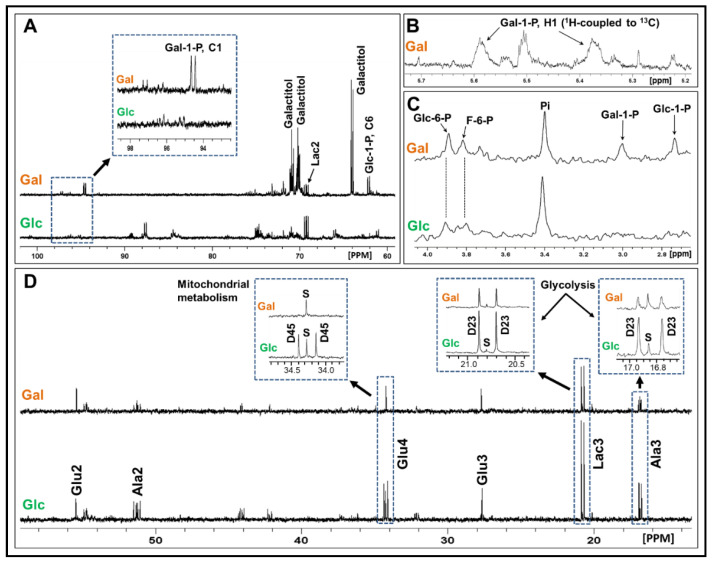
Multinuclear NMR spectroscopy data supports the presence of an active Leloir pathway in GBM cells. (**A**) Portions of the representative ^13^C NMR spectra of GBM cells treated with either [U-^13^C]Glc (lower) or [U-^13^C]Gal (upper), showing the C_1_ carbon of Gal-1-P (inset). The presence of this metabolite is evidence of the active Leloir pathway in GBM cells. Galactitol was also detected in Gal-treated GBM cells, suggesting the presence of aldose reductase activity in GBM cells. (**B**) ^1^H NMR signal of H-1 of the Gal-1-P in [U-^13^C]Gal-treated GBM cells, showing the ^1^H-^13^C spin–spin coupling in Gal-1P. (**C**) ^31^P NMR signals of Glc-1-P and Gal-1-P in Gal-treated GBM cells (upper panel) and Glc-treated cells (lower panel). (**D**) Portions of the ^13^C NMR spectra of GBM115 cells treated with [U-^13^C]Gal (upper) and [U-^13^C]Glc (lower). Insets showing Glu4, Lac3, and Ala3 from both Gal- and Glc-treated cells.

To gain a better understanding of the metabolism of Gal by GBM, we undertook an investigation to determine the metabolic fate of Glc and Gal using ^13^C-labeled sugars ([U-^13^C]Glc or [U-^13^C]Gal) and ^13^C metabolic flux analysis [36]. Four primary GBM cell lines were used for this analysis. Figure 4A shows the ^13^C NMR spectra (60–100 ppm region) of extracts of GBM115 cells incubated with either [U-^13^C]Glc or [U-^13^C]Gal. The ^13^C signal observed at 94.55 ppm can be attributed to the formation of Gal-1-P from Gal, which clearly indicates the presence of an active Leloir pathway in these GBM cells. To further confirm the presence of an active Leloir pathway in GBM cells, we employed ^1^H and ^31^P NMR spectroscopy techniques. In the ^1^H NMR spectrum of [U-^13^C]Gal incubated cells, we detected the presence of ^13^C satellites of the H-1 proton of the Gal-1-P at 5.38 and 5.59 ppm (^1^J_CH_ = 172.39 Hz), which confirmed the presence of ^13^C-labeled Gal-1-P (Figure 4B). The ^31^P NMR spectrum of the Gal-treated GBM cells also showed the presence of Gal-1-P at 3.0 ppm and Glc-1-P at 2.74 ppm (Figure 4C). These sugar phosphates were not detected in Glc-treated GBM cells, which further confirms the presence of Gal metabolism in GBM cells via the Leloir pathway. The ^1^H, ^13^C, and ^31^P chemical shift assignments of Gal-1-P are in agreement with the literature data [37,38,39].

Figure 4D shows the portion of ^13^C NMR spectra of extracts of GBM115 cells incubated with [U-^13^C]Glc (bottom trace) and [U-^13^C]Gal (top trace). They show signals arising from the glycolytic and tricarboxylic acid (TCA) cycle intermediates of [U-^13^C]Glc and [U-^13^C]Gal metabolism in GBM cells. [U-^13^C]Glc is converted to [U-^13^C]pyruvate, via glycolysis, which in turn is converted to [U-^13^C]lactate by lactate dehydrogenase and [U-^13^C]alanine by alanine aminotransferase [36].

In the case of [U-^13^C]Gal incubated cells, Gal is converted to Gal-1-P and then to Glc-1-P, via the Leloir pathway, and is later converted to Glc-6-P by PGM1/PGM2 (Figure 1). Gal-derived Glc-6-P enters the glycolysis/TCA cycle similar to Glc metabolism. The C3 ^13^C signals of the glycolytic metabolites, lactate (Lac3) and alanine (Ala3), were detected at 20.8 and 16.88 ppm, respectively, in both [U-^13^C]Glc and [U-^13^C]Gal incubations. [U-^13^C]pyruvate further converted to [1,2-^13^C]acetyl-CoA by pyruvate dehydrogenase, which enters the TCA cycle. In the first turn of the TCA cycle, [1,2-^13^C]acetyl-CoA combines with unlabeled oxaloacetate and forms [4,5-^13^C]α-ketoglutarate/glutamate, showing as the doublet D45 in the C4 glutamate signal (Glu4) at 34.20 ppm (Figure 4D). The D45 signal is a readout of [1,2-^13^C]acetyl-CoA generated during [U-^13^C]Glc or [U-^13^C]Gal metabolism in the TCA cycle.

From Figure 4D, we can see that [U-^13^C]Gal is mostly converted to lactate (Lac3) and alanine (Ala3), and it is not entering the TCA cycle at any significant rate (absence of D45 signal of Glu4 in the inset). Moreover, relatively lower ^13^C-enrichment of Lac3 in Gal-treated GBM cells (78.0 ± 2.5% of that observed in Glc-treated cells) indicates a slightly reduced glycolytic flux in the Gal-treated cells. Supplemental Table S1 shows the relative fluxes of the ^13^C-labeled Glc and Gal, via the glycolysis and mitochondrial metabolism of four GBM cell lines. Although the glycolytic flux is comparable or slightly lower in [U-^13^C]Gal-treated GBM cells than in [U-^13^C]Glc-treated cells, the [1,2-^13^C]acetyl-CoA levels are significantly lower (0.4–0.6%) in [U-^13^C]Gal-treated cells compared to [U-^13^C]Glc-treated cells (6.8–19.2%). 

**Figure 5:**
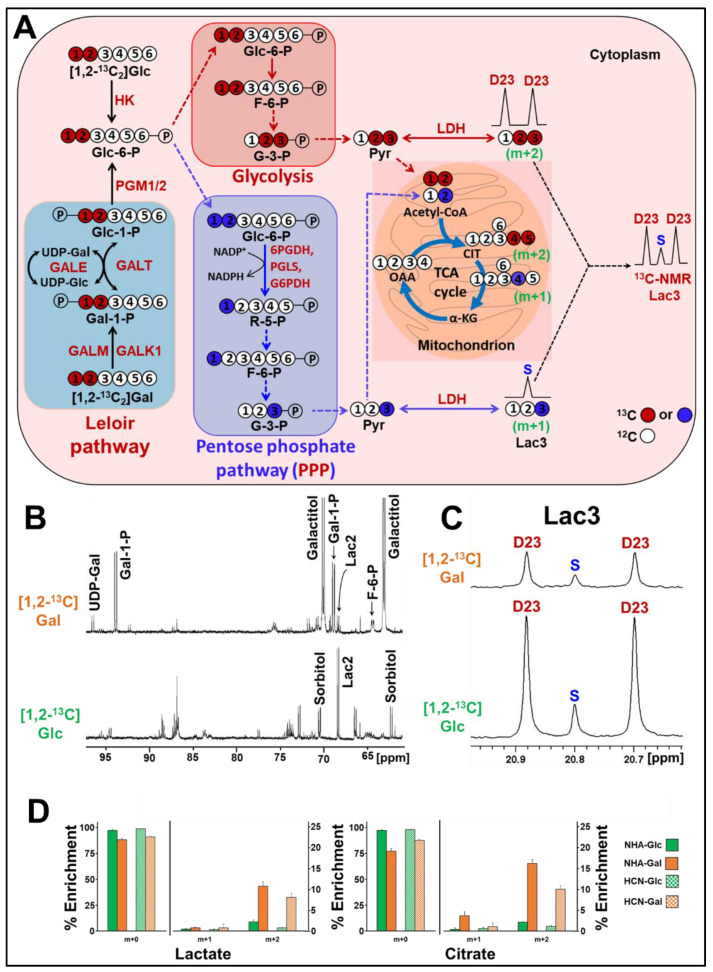
NMR and mass spectrometry together provide evidence for active glycolysis, Leloir pathway and PPP in Glc and Gal treated GBM and control cells (**A**) Schematic diagram showing the metabolism of [1,2-^13^C]Glc and [1,2-^13^C]Gal in GBM cells via glycolysis and PPP pathways. (**B**) ^13^C NMR spectra of GBM115 cells treated with [1,2-^13^C]Gal (upper) or [1,2-^13^C]Glc (lower), showing the Leloir pathway intermediates. (**C**) ^13^C NMR spectra showing the multiplet signals of Lac3. (**D**) Levels of carbon enrichments of lactate and citrate isotopomers obtained by GC-MS in NHAs and HCNs treated with [1,2-^13^C]Glc and [1,2-^13^C]Gal.

We observed an apparent shift in ^13^C-flux away from the canonical glycolysis/TCA cycle in Gal-treated compared to Glc-treated cells. The ^13^C signals of the phosphorylated carboxylic acids from the glycolysis and phosphorylated sugars are generally observed in the region 70–76 ppm [40]. The peak intensities of the ^13^C signals in this particular region suggest that the ^13^C-flux is partially shunted away from glycolysis into the pentose phosphate pathway (PPP) in Gal-treated GBM cells (Figure 4A). To test this hypothesis, we performed another series of experiments with GBM115 cells using sugars ^13^C-labeled only in the positions 1 and 2 carbons, [1,2-^13^C]Glc and [1,2-^13^C]Gal. Figure 5A illustrates the flow of carbons during the metabolism of [1,2-^13^C]Glc and [1,2-^13^C]Gal, including the PPP. Figure 5B shows the comparison of the ^13^C NMR spectra of GBM cells treated with [1,2-^13^C]Gal and [1,2-^13^C]Glc. The two key metabolic intermediates of the Leloir Pathway, Gal-1-P and UDP-Gal, were detected as ^13^C-labeled doublets of their C1 carbons at 94.58 ppm and 97.18 ppm, respectively. The ^13^C chemical shifts of these two key intermediates (Gal-1-P and UDP-Gal) are in agreement with the literature values [37]. The presence of these ^13^C signals was observed only in Gal-treated GBM cells, confirming Gal metabolism in GBM cells through the Leloir pathway. Next, we quantified the relative flux through the oxidative branch of the PPP with respect to glycolysis using the ^13^C multiplet signal of [2,3-^13^C]lactate at 20.8 ppm derived from [1,2-^13^C]Glc and [1,2-^13^C]Gal metabolism (Figure 5A,C). [2,3-^13^C]lactate produced by glycolysis appears as a doublet (D23), whereas [3-^13^C]lactate generated via PPP is seen as singlet (S) in the Lac3 ^13^C signal (Figure 5C). The ratio of singlet (S) to doublet (D23) of the Lac3 multiplet signal directly gives the relative flux through the oxidative branch of the PPP with respect to glycolysis [41]. In GBM115 cells treated with [1,2-^13^C]Gal, the ratio (S/D23) was determined to be 20% (*p* = 0.014) higher when compared to the [1,2-^13^C]Glc treatment (Figure 5C). This clearly suggests that the PPP flux is significantly higher in Gal-treated GBM cells.

To determine whether PPP is elevated in normal healthy astrocytes (NHAs) and human cortical neurons (HCNs) during Gal metabolism, we performed the above analysis using [1,2-^13^C]Glc and [1,2-^13^C]Gal in NHAs and HCNs. Since these cells grow very slowly, we used mass spectrometry-based ^13^C metabolic flux analysis. Figure 5D shows levels of the M + 0, M + 1, and M + 2 isotopologues of lactate and citrate detected during [1,2-^13^C]Glc and [1,2-^13^C]Gal metabolism in NHAs and HCNs. As shown in Figure 5A, the glycolytic pathway generates M + 2 whereas the PPP generates M + 1 isotopologues of lactate. Both NHAs and HCNs showed significant enrichment of M + 2 lactate in [1,2-^13^C]Gal treatment (10.71% and 8.05% in NHAs and HCNs, respectively) and showed relatively lower enrichment in the [1,2-^13^C]Glc treatment (2.21% and 0.77% in NHAs and HCNs, respectively). On the other hand, the level of the M + 1 lactate was <1% in both NHAs and HCNs during [1,2-^13^C]Glc and [1,2-^13^C]Gal metabolism (Figure 5D). These data clearly indicate that NHAs and HCNs metabolize Glc and Gal preferably through the glycolytic route than the PPP. Further, mitochondrial oxidation of [1,2-^13^C]Glc and [1,2-^13^C]Gal showed consistent ^13^C labelling patterns in the citrate, as shown in Figure 5D.

We noted another major metabolic difference in the GBM cells treated with [1,2-^13^C]Glc and [1,2-^13^C]Gal. The total peak areas of the ribose sugar units of the ATP and ADP combined was determined from the ^1^H NMR signals resonating between 6.12 ppm and 6.15 ppm. The peak area of the GBM cells treated with [1,2-^13^C]Gal was 2.4 times greater than the cells treated [1,2-^13^C]Glc (Figure S3E). This increase in the ATP/ADP pool size is presumably driven by elevated ribose-5-phosphate (R-5-P), an intermediate of the PPP, as R-5-P is the rate-limiting substrate of ribose-phosphate diphosphokinase (Figure 5A).

We also quantified the mRNA transcript levels of the enzymes involved in Gal metabolism in various human tissues (brain, liver, pancreas, and heart) using the GTEx portal (https://gtexportal.org/home/, accessed on 16 February 2021). Among these tissues, the liver showed higher levels of Leloir pathway gene transcripts (GALM, GALK1, GALT, GALE, PGM1, and PGM2). Brain tissue showed low to moderate levels of Leloir pathway enzymes, with high levels of PGM2L1 (Supplemental Figure S3A). Next, we tested if normal brain has an active Leloir pathway by isolating brain cells from a wild-type mouse and tested their oxygen consumption in the presence of Glc and Gal. Incubation of brain cells in the presence of Glc increased the respiration by 25% whereas incubation in the presence of Gal decreased the oxygen consumption by 35% (Figure S3B).

To determine the relative metabolic flux in normal brain cells, we incubated mouse brain cells with [U-^13^C]Gal or [U-^13^C]Glc for 2 h and determined the total ^13^C-lactate (Lac3) levels in both groups. The levels were significantly lower (11%) in Gal-treated brain cells compared to Glc-treated cells. A small amount of ^13^C-alanine (Ala3) was also detected in Glc-treated cells while no Ala3 was detected in Gal-treated cells (Figure S3C). Consistent with our earlier GBM cells data, we only detected Glut4 in [U-^13^C]Glc-treated brain cells, and the absence of Glut4 in cells incubated with [U-^13^C]Gal suggest that Gal is not entering the mitochondria (Figure S3D). This observation is in line with the decreased oxygen consumption by mouse brain cells in the Gal treatment (Figure S3B), as discussed above.

Finally, to assess the impact of elevated UDP-Gal/UDP-Glc (a consequence of a functioning Leloir pathway), we examined the impact of hexose substitution on glycan expression. GBM115 cells were incubated with 4 mM Glc, 2 mM Glc and 2mM Gal (Glc&Gal), or 4 mM Gal, and after 48 h, the cells were fixed in 4% paraformaldehyde (in PBS) and were labeled with FITC-conjugated *Bauhinia purpurea* agglutinin (BPA), peanut agglutinin (PNA), and concanavalin A (ConA) lectins as fluorochrome probes:

(i.) BPA binds to the T-antigen (Galß(1-3)GalNAc).
(ii.) PNA binds to glycans with a terminal galactose.
(iii.) ConA binds to mannose and shows the levels of both immature and mature N-glycans.

**Figure 6:**
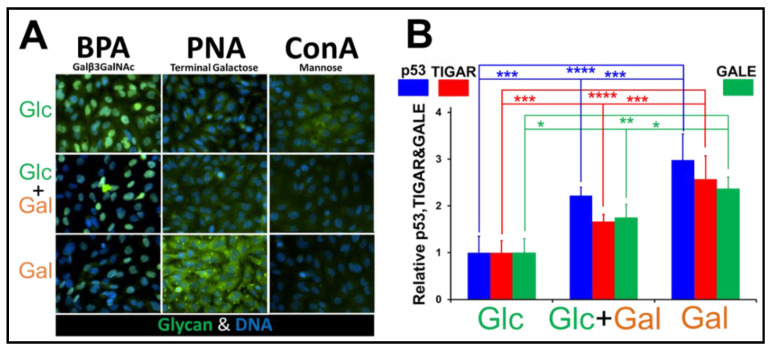
Glycan labeling and levels of p53/TIGA/GALE in Glc, Glc&Gal, and Gal treated GBM cells. (**A**) The labeling of glycans in GBM115 cells grown on Glc, Glc and Gal, or Gal (48 h). The glycans were labeled with FITC-lectins (green) and their nuclei were labeled with DAPI (blue). (**B**) Graph showing the levels of p53, TIGAR, and GALE as a function of increased Gal in GBM cells treated with Glc, Glc and Gal, or Gal alone (mean ± SEM; * *p* < 0.05, ** *p* < 0.001, *** *p* < 0.005 and **** *p* < 0.0001. *n* = 8 (p53 and TIGAR) and *n* = 4 (GALE). Measured by immunofluorescence microscopy.

Figure 6A shows the labeling of glycans in GBM115 cells grown on Glc, Glc&Gal, or Gal with FITC-lectins. When Glc was gradually replaced by Gal, we observed a collapse in the BPA labeling of T-antigen (Galß(1-3)GalNAc), an increase in galactose-capped glycans by PNA, and a drop in high mannose glycan labeling by ConA (Figure 6A). The mismatch between the supply and demand for UDP-Gal/UDP-GalNAc triggers mild ER stress and causes pathway activation and the elevation in the levels of TIGAR. An increase in levels of p53, TIGAR, and GALE was also observed when the concentration of Gal was increased in the media (Figure 6B), which could be due to triggering of the unfolded protein response (UPR). These observations further confirm the ER stress in Gal-incubated GBM cells.

## 3. Discussion

The upregulation of Glut3 in GBM and its correlation with poor patient outcomes have been documented by others [9,42,43]. In this study, we demonstrated that its nominally testicular-specific paralogue, Glut14, is also expressed in GBM, and this expression correlates with poor outcomes. Kinetics analysis indicated that Glut3 and Glu14 support high rates of Gal transport and growth studies showed that GBM cells proliferate well on Gal. When Glut3/Glut14 hexose transport is inhibited by antibody binding, we found a greater impact on GBM cell growth with Gal compared to Glc.

This shift from glycolysis into the PPP was validated and quantified using [1,2-^13^C]Glc/[1,2-^13^C]Gal stable isotope tracer-based ^13^C NMR metabolic studies and indicated a quite unexpected usage of the PPP by GBM, to generate key carbon precursors for macromolecular synthesis and NADPH. These results contrast with a previous study that showed that PPP was not elevated in mouse models of GBM and brain metastases, compared with non-tumor parts of the brain [41].

It is often stated in the literature that, while the metabolism of Glc to lactate yields two net ATP per hexose, the combined Leloir/glycolysis pathway yields net zero ATP during the conversion of Gal to pyruvate, and as a result, mammalian cells grown on Gal have an absolute reliance on oxidative phosphorylation for ATP synthesis (see [44] and references therein). However, the yield of ATP from the anaerobic metabolism of Gal to lactate is also two per hexose, and the usage of a molecule of UDP-Glc for the conversion of Gal-1-P into UDP-Gal is catalytic and thus not a metabolic sink. Moreover, blocking glycolysis at PFK, which results in the elevation of both F-6-P and G-6-P, causes a diversion of carbon flux into the PPP and the hexosamine pathways. Utilizing the energy derived from hexose oxidative decarboxylation of the PPP allows cells to eke out slightly more energy from the anaerobic metabolism of Gal into lactate than in the case of Glc oxidation via glycolysis:

This indicates an unexpected usage of the PPP by GBM, maximizing ATP production during Gal oxidation, from some of the energy derived from the (irreversible) oxidative decarboxylation of 6-phosphogluconate. We show the passage of carbon, from [1,2-^13^C] labeled hexoses, into the four main flux pathways: glycolysis, Leloir, PPP, and the hexosamine pathway. Presenting GBM cells with high levels of Gal will cause an elevation in UDP-Gal, and the conversion into UDP-Glc will monopolize GALE. In addition to being vital for the Leloir pathway, GALE is also critical for the generation of UDP-GalNAc from UDP-GlcNAc in the hexosamine pathway [45]. Competitive inhibition, at GALE, between the Leloir (UDP-Gal) and hexosamine (UDP-GlcNAc) pathways would mean that the ER/Golgi will receive an excess of UDP-Gal and a deficit of UDP-GalNAc. This was noted by using the lectins PNA and BPA, which showed an elevation of Gal-tipped glycans and the loss of the T-antigen, Galß(1-3)GalNAc (Figure 6). The mismatch between supply and demand for UDP-Gal/UDP-GalNAc triggers mild ER stress and causes pathway activation and the elevation in the levels of TIGAR. TIGAR consumes F-2,6-P2, and so halts the activity of PFK; this, in turn, elevates the levels of both F-6-P and Glc-6-P, whose elevation was observed using ^31^P and ^13^C NMR (Figure 4C and Figure 5B). The elevation of F-6-P will increase carbon flux into the hexosamine pathway, increasing [UDP-GalNAc] and partially overcoming the competition for GALE between the Leloir/hexosamine pathways. Physiological stresses, including glucose starvation, hypoxia, increased temperature, and an interruption in the supply of UDP-hexoses, activate the ER sensors, ATF6, PERK, and IRE1. ER stress signaling, via PERK, can lead to increased levels of TIGAR (TP53-induced glycolysis and apoptosis regulator) via increases in canonical p53 and the p53/p47 variant [46,47,48,49]. Additionally, ER stress can activate the IRE1/Xbp1s signaling cascades and elevate the Leloir pathway enzymes GALE and GALK [50]. We measured the levels of p53, TIGAR, and GALE in GBM cells incubated for 48 h in 4 mM Glc, 2 mM Glc and 2mM Gal, or 4 mM Gal. All three markers of ER stress signaling were elevated in the presence of Gal (Figure 6B). Increases in the three proteins suggest GBM cells use ER stress as a means of galactose sensing and their metabolic plasticity derives from this signaling network.

When Gal is given to the GBM, it is taken up by Glut3/Glut14 and metabolized into UDP-Gal, which is then converted into UDP-Glc by GALE, before being converted into Glc-6-P. GALE is not only used by the Leloir pathway but also by the hexosamine pathway, where it is required to convert UDP-GlcNAc into UDP-GalNAc [45]. When Gal is metabolized by the Leloir pathway, the UDP-Gal/UDP-Glc levels must become elevated. This elevation will saturate GALE and slows down the conversion of UDP-GlcNAc into UDP-GalNAc. The lack of UDP-GalNAc will disrupt glycan maturation and lead to ER stress, trigging the unfolded protein response (UPR), which increases the levels of p53, TIGAR, and GALE. TIGAR converts F-2,6-P2 into F-6-P, and removes the activation of PFK, halting glycolysis at this level and increasing both F-6-P and Glc-6-P. The elevation of the F-6-P levels will increase the steady-state levels of UDP-GlcNAc. The Glc-6-P is diverted into the PPP, and undergoes oxidative decarboxylation, generating NADPH, F-6-P, and G-3-P. The F-6-P is in dynamic equilibrium with Glc-6-P and can either reenter the PPP or continue past the mostly inhibited PFK and converted into G-3-P, and hence leads to the formation of lactate. Not only does this diversion of carbon into the PPP allow efficient Gal metabolism that generates ATP from ADP, but also it serves to increase levels of NADPH. The overall thermodynamic efficiency of using the PPP for the oxidation of Glc-6-P into lactate/CO_2_ is far lower if channeled into oxidative phosphorylation as tumor/cancer cells rarely have an adequate supply of oxygen to maintain mitochondrial carbon flux.

Although we used patient-derived primary GBM cells in this study, the use of stem-like cells from freshly resected GBM tumors would provide a better insight in understanding Gal metabolism in GBM. Although this is a limitation in the current study, we are planning for such studies in the future. Moreover, the in vivo Gal metabolism studies in GBM patients will be critical to elucidate metabolic networks and identify the key metabolic pathways. In this regard, we have initiated in vivo metabolism studies in brain tumor patients (GBMs and meningiomas) by infusing non-radioactive ^13^C-labeled nutrients, such as Glc and acetate, 2 h prior to the surgical resection of the tumor mass. The tumor tissue collected during the surgery has been used to investigate the in vivo metabolism of Glc/acetate in brain tumors by ^13^C-NMR-based isotopomer analysis [6]. We will perform similar studies using ^13^C-Gal compounds as metabolic tracers to investigate the in vivo metabolism of Gal in GBM patients.

## 4. Materials and Methods

### 4.1. Chemicals

Unless otherwise indicated, all reagents and antibodies were sourced from Sigma-Aldrich (Sigma-Aldrich, St. Louis, MO, USA). [U-^13^C]Glc, [U-^13^C]Gal, perchloric acid, KOH, and phenylphosphonic acid (PPA) were purchased from Millipore Sigma (Miamisburg, OH/St. Louis, MO, USA). D_2_O, DCl, and NaOD were purchased from Cambridge Isotope Laboratories (Tewksbury, MA, USA).

### 4.2. Tumor Tissues and Cell Lines

All 5 cell lines (GBM111, GBM115, GBM133, GBM157, and GBM175) are Grade IV GBM cells and were generated from the tumor tissue specimens collected in the operating room from patients with GBM undergoing surgical resection of the tumor. Other genetic and molecular signatures are given below: All the patient tumors were classified as GBM (WHO 2007 Grade IV) by clinical neuropathologists at our hospital. All tumors were the IDH1 wildtype. For GBM115, MGMT methylation status was found to be unmethylated and no EGFR amplification was detected. The GBM133 patient was GFAP positive and showed the following genomic alterations: PDGFRA and MDM4 amplification and PIK3R1 R461_E462 > Q mutation. GBM157 was a recurrent GBM patient. Tumor tissues were washed with cold PBS (Fisher Scientific, Waltham, MA), minced with a scalpel, homogenized, and grown in Dulbecco’s modified Eagle’s medium (DMEM) with fetal bovine serum (FBS, 20%), 1U GlutaMax™, sodium pyruvate (1mM), penicillin (100 U/mL), and streptomycin (100 mg/mL). All GBM cells (GBM111, GBM115, GBM133, GBM157, and GBM175) used in this study were spontaneously immortalized and primary stocks were frozen at the fourth or fifth passage. For the current study, cells from seventh to ninth passages were used. Human gallbladder and testicle tissues were obtained from the Houston Methodist Hospital biorepository. In one of the patients, a small amount of normal brain tissue was removed during the surgery to reach to the tumor and was used as a normal control tissue. The mice tissue specimens were obtained from wild-type mice following institutional animal care and use committee (IACUC) Protocol AUP-0315-0016, approved by the IACUC of Methodist Hospital. Control cell lines (NHA and HCN) were purchased from ATCC (American Type Culture Collection, Manassas, VA, USA).

### 4.3. Patient Survival Data and Statistical Analysis

The Cancer Genome Atlas Glioblastoma dataset (TCGA-GBM; RNA Seq V2 RSEM), was examined to determine the transcript levels of genes and patient overall survival (months; OS) [27]. The examined cohort consisted of 200 patients with <83 months OS. Optimal high/low transcript cutoffs for the generation of Kaplan–Meier survival curves were identified from maximum difference of ranked means and statistical significance calculated using Cox–Mantel log-rank Chi-square tests [28],

Kaplan–Meier survival curves and analysis of the transcript levels obtained from three databases, TCGA-GBM Agilent-4502A [29], Rembrandt [30], and Gravendeel [31] (Supplemental Figure S2B), were generated using GlioVis (http://gliovis.bioinfo.cnio.es/, accessed on 16 February 2021).

### 4.4. Immunohistochemistry

Immunohistochemical analysis of Glut3-rich human gallbladder and human brain alongside human testicular tissue were done using the ab15311 and HPA006539 antibody pair.

### 4.5. Fluorescence Microscopy

Images were captured using a Nikon Eclipse TE2000-E using a CoolSnap ES digital camera system (Nikon Instruments Inc., Melville, NY, USA). Primary antibodies were visualized using Alexa-594 donkey anti-mouse and anti-rabbit antibodies. FITC-labeled lectins were obtained from EY Laboratories (San Mateo, CA, USA).

### 4.6. Cell Viability and Growth Studies

Growth studies were performed to test the hypothesis that primary GBM can readily metabolize Gal, performed using Gal as the sole energy source utilizing five primary GBM cell cultures (GBM111, 115, 133, 157, and 175).

Cells grown in 96-well plates were incubated for 30 min with Hoechst 33342 (10 µM) and then fixed with 4% paraformaldehyde. Cell counts were performed on images taken of the center field. Dead/dying cells were identified as having condensed nuclei with signal intensities over three-fold that of the median cell nuclei or being identified as fragmented.

### 4.7. Transporter Modeling

The classical Michaelis–Menten inhibitor equation was used to model competition kinetics: *v* = ((V_max_*[S]*K_i_))/(K_m_ + [S])(K_i_ +[I])), which was fitted at four fixed Gal concentrations, from 0 to 6 mM Glc. For Glut1 the K_m_ for Gal of 17 mM and a Glc K_i_ of 0.4 was used, where Gal V_max_ was 66% Glc V_max_. For Glut3 the K_m_ for Gal of 8.5 mM and a Glc K_i_ of 6.9 was used, where Gal V_max_ was 120% Glc V_max_.

### 4.8. NMR Experiments

#### 4.8.1. GBM Cell Culture and Perchloric Acid Extraction

DMEM (Glc-free) media containing (1) 6.0 mM [U-^13^C]Glc/[1,2-^13^C]Glc or (2) 6.0 mM [U-^13^C]Gal/[1,2-^13^C]Gal was added to the washed GBM cultures. After 5 h of incubation, the medium was removed, and cells were washed with PBS, harvested, and the cell pellets were snap-frozen. Cell pellets were extracted in 5% perchloric acid, centrifuged, neutralized, and extracts dried in a CentriVap^®^ vacuum concentrator (Labconco Corporation, Kansas City, MO, USA). Residues were reconstituted in 180 µL D_2_O containing 1.0 mM DSS-d_6_ (internal standard), and the pH adjusted to 7.4 ± 0.05.

#### 4.8.2. Metabolism of [U-^13^C]Gal and [U-^13^C]Glc in Normal Mouse Brain Cells

Brain cells were extracted from a wild-type mouse by homogenizing the mouse brain in a tissue homogenizer (BeadBug^TM^, Benchmark Scientific, Edison, NJ, USA). The brain cells were washed with 0.9% saline and were incubated with (1) 6.0 mM [U-^13^C]Glc or (2) 6.0 mM [U-^13^C]Gal prepared in 0.9% saline. After 2 h, the hexose medium was removed and cells were washed with PBS, harvested, and the cell pellets were snap-frozen. The cell pellets were extracted in 5% perchloric acid and samples were prepared for NMR experiments as explained in the above section.

#### 4.8.3. ^1^H and ^13^C NMR Experiments

Proton (^1^H) and ^1^H-decoupled ^13^C NMR data were acquired on a Bruker NMR spectrometer (Bruker Biospin, Billerica, MA, USA) operating at 600/800 MHz (^1^H frequency) or 150/201 MHz (^13^C frequency), equipped with a cryogenically cooled ^13^C direct detection probe. The ^1^H NMR spectral data were collected by using the nuclear Overhauser effect (NOE) pulse sequence with suppression of the water signal. ^1^H NMR data were collected by a 90° pulse excitation pulse (duration = 10 µs) with 128 scans, 132 k time domain points and with a relaxation delay of 2.0 s. The spectral width was 9615 Hz and the acquisition time was 1.70 s; mixing time for the NOE was 100 ms. ^1^H NMR time domain data were multiplied by an exponential window function (line broadening = 0.3 Hz) before Fourier transformation. The resulting spectra were referenced to the internal standard DSS-d_6_ (0 ppm). A power-gated sequence with a WALTZ-16 composite pulse (flip-angle = 30°) was used to acquire the ^1^H-decoupled ^13^C NMR data. The acquisition parameters used were as follows: number of scans = 10,000; 90° pulse = 10 µs; time domain points = 144 k; interpulse delay = 2.5 s; spectral width = 36,058 Hz; acquisition time = 2.0 s, and line broadening for the exponential window function = 1.0 Hz. The lactate C3 carbon signal at 20.8 ppm was used as an internal chemical shift reference. Peak areas of various ^13^C signals were measured by deconvolution using ACD software (Advanced Chemistry Development, Toronto, ON, Canada).

#### 4.8.4. ^13^C NMR Isotopomer Analysis

^13^C NMR isotopomer analyses were performed as reported in our earlier publications [6,7,36]. Briefly, [U-^13^C]Glc is converted to [U-^13^C]pyruvate, via glycolysis, which in turn is converted to [U-^13^C]lactate by lactate dehydrogenase and [U-^13^C]alanine (Figure 4D showing lactate C3 signal (Lac3) at 20.80 ppm and alanine C3 signal (Ala3) at 16.88 ppm. In addition, [U-^13^C]pyruvate is also converted to [1,2-^13^C]acetyl-CoA by pyruvate dehydrogenase and then enters the TCA cycle. In the first turn of the TCA cycle, [1,2-^13^C]acetyl-CoA combines with unlabeled oxaloacetate and forms [4,5-^13^C]α-ketoglutarate/glutamate (Figure 4D showing Glutamate C4 signal (Glu4) at 34.20 ppm). ^13^C-labeled lactate (Lac3), and glutamate (Glut4) can be readily detected by ^13^C NMR spectroscopy and were used for ^13^C isotopomer analysis [6,7,36]. The normalized peak areas of the ^13^C-^13^C doublet (D23) and singlet (S) of Lac3 were obtained from the ratio of the peak area of the corresponding multiplet component (D23 or S) to the total area of the Lac3 signal (the sum of the peak areas of D23 and S). Similarly, the normalized peak area of the ^13^C-^13^C doublet (D45) or the singlet (S) signal of Glu4 was obtained from the ratio of the peak area of the corresponding multiplet component (D45 or S) to the total area of Glu4 signal (the sum of the peak areas of D45 and S). On the other hand, [U-^13^C]Gal is converted to [U-^13^C]Gal-1-P and was accumulated in GBM cells while a small fraction of [U-^13^C]Gal-1-P was isomerized to [U-^13^C]Glc-1-P by the action of GALT. [U-^13^C]Glc-1-P was further metabolized to [U-^13^C]Glc-6-P and entered the glycolysis pathway to produce [U-^13^C]pyruvate and further metabolized to Lac3, Ala3, and/or Glu4, similar to the [U-^13^C]Glc metabolism as discussed above.

In the cells treated with [1,2-^13^C]Glc or [1,2-^13^C]Gal, the ^13^C isotopomer analysis was performed as described in the literature [41].

#### 4.8.5. ^31^P NMR Experiments

^31^P NMR experiments were performed on a Bruker 800 MHz NMR spectrometer (^31^P resonance frequency of 323.90 MHz). A 5.0 mM PPA solution was used as an external reference (14.2 ppm). The ^1^H-decoupled ^31^P NMR spectra were obtained by using a power-gated sequence with a 30° flip-angle and with a WALTZ-16 composite pulse. The following acquisition parameters were employed: number of scans = 500, 90° pulse = 36.8 µs, number of points in time domain = 32 k, interpulse delay = 5 s, spectral width = 12,931 Hz, acquisition time = 1.27 s, and line broadening for the exponential window function = 3.0 Hz.

#### 4.8.6. [1,2-^13^C]Glc/Gal Metabolism Studies in NHAs and HCNs

NHAs were grown in ABM^TM^ (Astrocyte Basal Medium), supplemented with AGM^TM^ (Astrocyte Growth Medium) SingleQuots^TM^ Supplements (FBS, L-glutamine, gentamycin/amphotericin, ascorbic acid, HEGF, and insulin) and incubated at 37 °C under humidified air with 5% CO_2_. Cells were grown in 100 mm × 20 mm culture dishes in replicates (*n* = 3) for each experiment. HCNs were grown in high-glucose DMEM (supplemented with 10% FBS, 4.0 mM L-glutamine). When both the NHAs and HCNs reached confluency, they were treated with isotopically labeled 6.0 mM [1,2-^13^C]Glc or 6.0 mM [1,2-^13^C]Gal and incubated for 24 h, harvested in 50% methanol, snap-frozen in liquid N_2_, and the lysates were stored at −80 °C.

#### 4.8.7. ^13^C isotopomer Analysis by GC-MS Analysis

For the ^13^C isotopomer analysis of intracellular metabolites by GC-MS, snap-frozen NHAs and HCNs were thawed and centrifuged to remove the protein precipitations. Sodium 2-oxobutyrate (50 nmol) was added to the supernatants as an internal standard. The samples were evaporated and derivatized by trimethylsilylation (Tri-Sil HTP, Thermo Scientific). A total of 3 µL of the derivatized solution was injected onto an Agilent 6970 gas chromatograph equipped with a fused silica capillary GC column (30-m length/0.25-mm diameter) coupled with an Agilent 5973 mass selective detector [51]. The measured distributions of the carbon isotopomers were corrected for the natural abundance of the ^13^C isotopomer (1.1%).

## 5. Conclusions

GBMs not only have highly efficient Gal importer proteins and the enzymes of the Leloir cycle, they also have the means of detecting the presence of Gal in their surroundings and to remodel their metabolism to best take advantage of this resource. We believe that this finding is novel, and that it provides insight into a new strategy to treat GBM by focusing on this metabolic pathway. Stem-like cells generated from the freshly resected GBM tumors and in vivo metabolism of Gal in GBM patients will be valuable to gain a better understanding and identifying the key metabolic pathways involved in Gal metabolism. Such studies would help in discovering the druggable targets to inhibit Gal metabolism in GBM patients. Gal-based antimetabolites, such as 4-deoxy-4-fluoro-D-galactose [52], can be used to interfere with the normal metabolic processes involved in the Leloir/PPP pathway in GBM cells. Further work on targeting this pathway using Gal-based antimetabolites is underway.

## Data Availability

The data presented in this study are available on request from the corresponding author.

## Supplementary Materials

The following are available online at https://www.mdpi.com/article/10.3390/cancers13081815/s1, Figure S1: Heatmap of genes involved in Leloir pathway, plasma galactose levels in healthy volunteers following galactose ingestion, Glut3 and Glut14 mRNA in male and female GBM, and correlation of Glut3 and Glut14 transcripts in GBM, Figure S2: Transcript levels of Glut14 in the TCGA-GBM Agilent 4502A database show that expression correlates with poor patient outcome. Figure S3: Heatmap of the relative levels of mRNA transcripts involved in Gal catabolism in human tissues (liver, pancreas, heart and brain), oxygen consumption, glycolysis and mitochondrial metabolism in isolated mouse brain cells in the presence of Glc or Gal. Table S1: ^13^C labeling of Lactate, Alanine, Glutamate and Acetyl-CoA in four GBM primary cell lines following incubation with ^13^C-Glc or ^13^C-Gal.
