# Supplementary material for: The Leloir Cycle in Glioblastoma: Galactose Scavenging and Metabolic Remodeling
Source: Cancers (Basel). 2021 Apr 10;13(8):1815. doi: 10.3390/cancers13081815 (PMC8069026; doi:10.3390/cancers13081815)
Supplement: Supplementary file 1 [file cancers-13-01815-s001.pdf]

cancers

Article

The Leloir Cycle in Glioblastoma: Galactose Scavenging and Metabolic Remodeling

Martyn A. Sharpe, Omkar B. Ijare, David S. Baskin, Alexandra M. Baskin, Brianna N. Baskin, Kumar Pichumani

Supplemental Figures

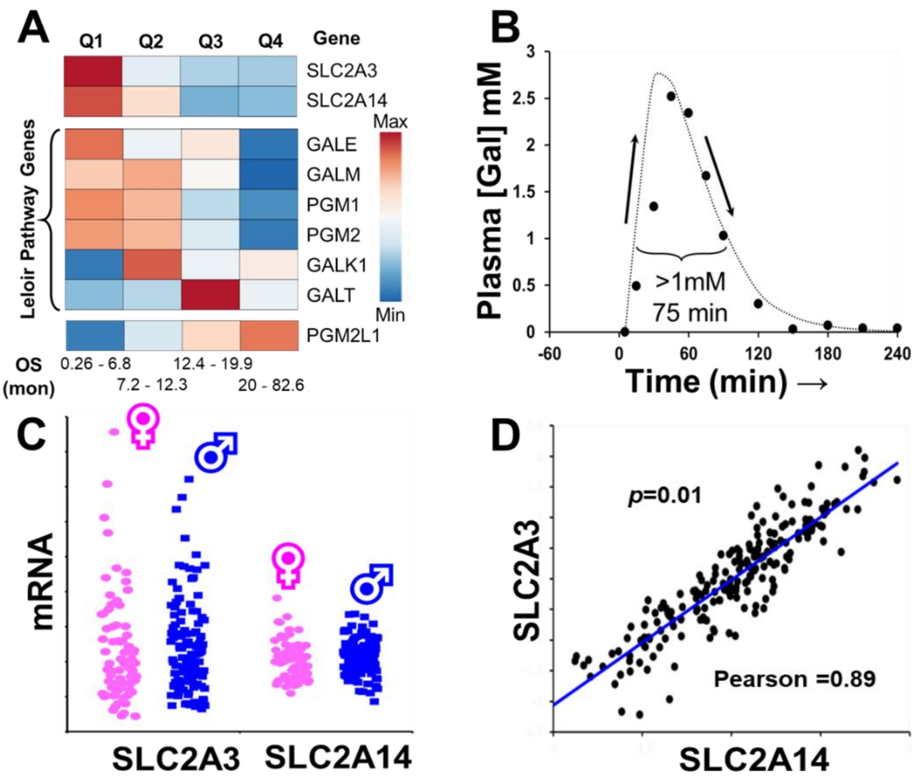

Figure S1. Correlation between key enzyme levels of the Leloir pathway and overall patient survival, plasma levels of Gal after ingestion in humans, and the transcript levels of the Gal transporters, SLC2A3 & SLC2A14 and their correlation. (**A**) Heatmap of genes involved in Leloir pathway (TCGA-RNASeq database) stratified by uncensored GBM patient overall survival (OS) in months. (**B**) Plasma galactose levels in healthy volunteers following galactose ingestion, (**C**) Glut3 and Glut14 mRNA in male and female GBM. Pink, female; Blue, male. (**D**) Correlation of Glut3 and Glut14 transcripts in GBM (TCGA RNA-Seq).

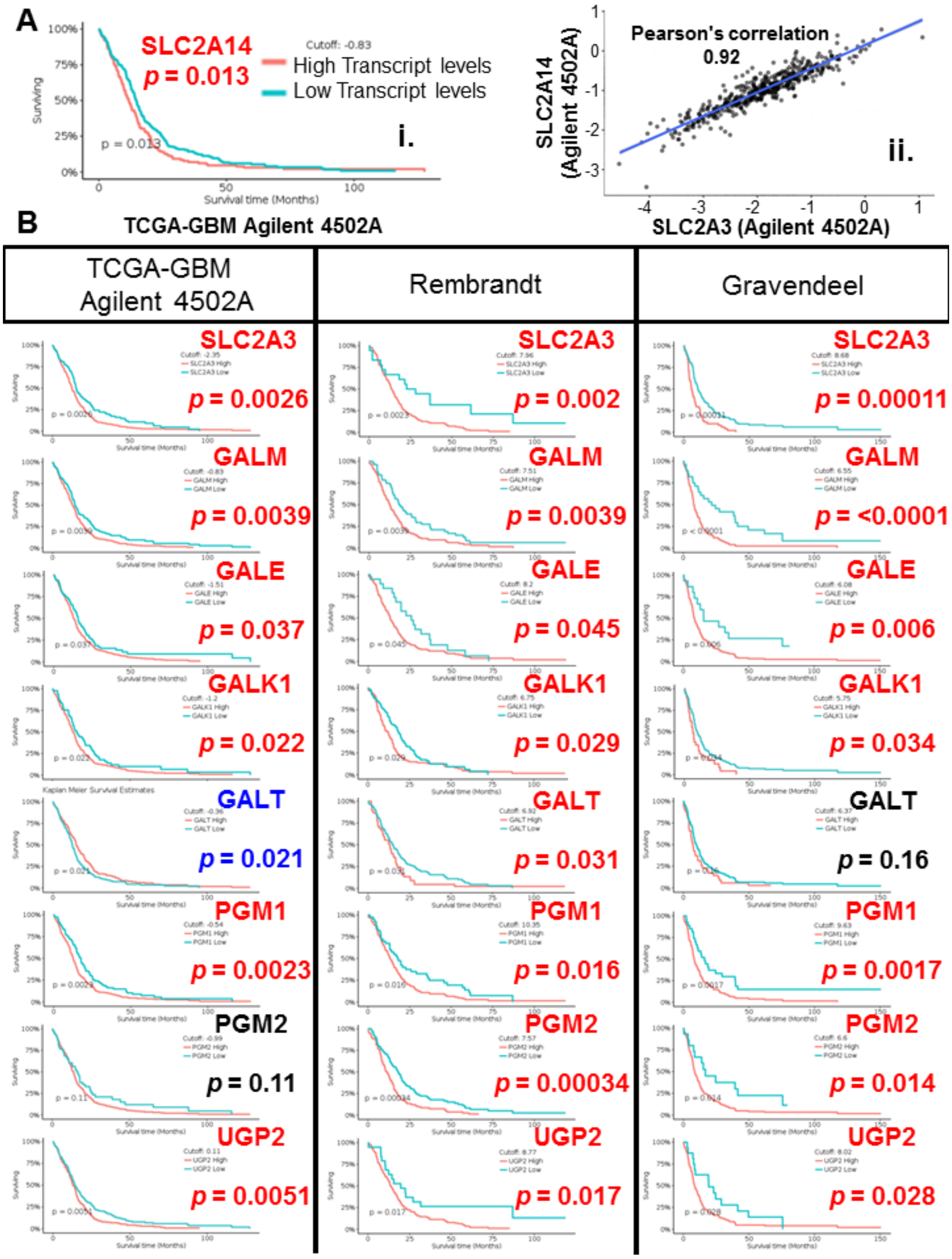

Figure S2: Transcript levels of Glut14 in the TCGA-GBM Agilent 4502A database show that expression correlates with poor patient outcome. Moreover, transcripts of SLC2A3 and SLC2A14 are highly correlated (**A**i and **A**ii). Using the GlioVis website, for brain tumor data analysis and visualization (http://gliovis.bioinfo.cnio.es/) we show the correlation between Gal catabolism gene transcript levels and patient outcome in three different GBM datasets (**B**).

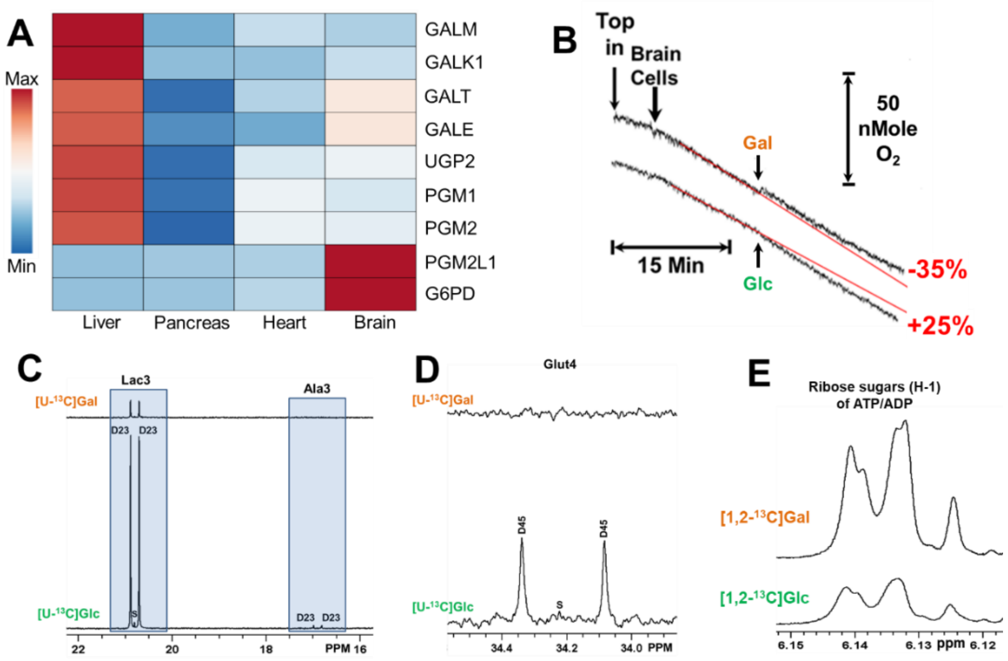

Figure S3: Transcript levels of Leloir pathway genes in different human tissues, oxygen consumption of mouse brain cells in the presence of Glc or Gal, and their glycolytic and mitochondrial metabolism of ^{13}C-Glc and ^{13}C-Gal. (**A**) Heatmap of the relative levels of mRNA transcripts (from GTEx portal) involved in Gal catabolism in four human tissues, Liver (classical Leloir), Pancreas, Heart and Brain shows that liver has high levels of Leloir pathway enzyme gene transcript, not present in other tissues. Additionally, brain has high levels of PGM2L1. (**B**) Representative oxygen electrode traces of mouse brain cells consuming 20 mM Glc or Gal. Addition of Glc to isolated brain cells increases respiration (by 25%), whereas addition of Gal causes a suppression of oxygen consumption (by 35%). (**C**) ^{13}C NMR examination of lactate (Lac3) and alanine (Ala3) (pyruvate) pools in Gal and Glc incubated mouse brain cells shows that the relative flux of Gal to lactate is only ≈11% than that observed with Glc. The levels of Ala3 in Gal incubated cells are zero (no ^{13}C signal), showing that no pyruvate is being generated from Gal. (**D**) The ^{13}C NMR regions of glutamate (Glu4) in Gal and Glc incubations show that there is no flux of Gal to glutamate (no ^{13}C signal), suggesting that there is no entry of Gal derived pyruvate/acetyl-CoA into the mitochondria. (**E**) Relative levels of Ribose sugars (from ATP/ADP) in [1,2-^{13}C]Gal (upper) and [1,2-^{13}C]Glc incubated GBM115 cells showing higher levels of ribose sugars in Gal incubations.

Table S1. ^{13}C labeling of Lactate, Alanine, Glutamate and Acetyl-CoA in four GBM primary cell lines following incubation with ^{13}C-Glc or ^{13}C-Gal.

GBM Line	Hexose/ Flux	Natural ^{13}C/Enriched ^{13}C*			[1,2-^{13}C] acetyl-CoA
		Lac S/D23	Ala S/D23	Glu S/D45	
175	^{13}C-Glc	0.14	0.37	0.19	19.2%
	^{13}C-Gal	0.20	12.20	2.09	0.6%
	Flux Glc/Gal	70%	3%	9%	32
157	^{13}C-Glc	0.19	0.19	0.16	15.2%
	^{13}C-Gal	0.20	1.32	13.40	0.5%
	Flux Glc/Gal	95%	14.4%	1.2%	30.4
111	^{13}C-Glc	0.12	0.35	0.36	11.9%
	^{13}C-Gal	0.07	1.06	3.71	0.8%
	Flux Glc/Gal	171.4%	33%	9.70%	14.9
115	^{13}C-Glc	0.05	0.16	0.35	6.8%
	^{13}C-Gal	0.07	0.49	11.28	0.4%
	Flux Glc/Gal	71.4%	32.6%	3.1%	17.0
Mouse Brain	^{13}C-Glc	0.013	0.04	0.35	103%
	^{13}C-Gal	0.014	nd	nd	nd
	Flux Glc/Gal	92.9%	-	-	-

*Smaller numbers mean greater turnover of the pool
